# Medical students as health coaches: Implementation of a student-initiated Lifestyle Medicine curriculum

**DOI:** 10.1186/s13584-017-0167-y

**Published:** 2017-11-10

**Authors:** Rani Polak, Adi Finkelstein, Tom Axelrod, Marie Dacey, Matan Cohen, Dennis Muscato, Avi Shariv, Naama W Constantini, Mayer Brezis

**Affiliations:** 1 Department of Physical Medicine and Rehabilitation, Institute of Lifestyle Medicine, Spaulding Rehabilitation Hospital, Harvard Medical School, 300 First Avenue, Charlestown MA, Boston, MA 02129 USA; 2 Department of Family Medicine, Hadassah-Hebrew University School of Medicine, Jerusalem, Israel; 3 Department of Nursing, Jerusalem College of Technology, Jerusalem, Israel; 4 “Adam U’Refuah” Program of Medical Humanities, Hadassah-Hebrew University School of Medicine, Jerusalem, Israel; 5 Department of Family Medicine and Braun School of Public Health‬‬‬‬‬‬‬‬‬‬, Hadassah-Hebrew University of Jerusalem‬‬‬‬‬‬‬‬‬‬, Clalit Health Care Services‬‬‬‬‬‬‬‬‬‬, Jerusalem, Israel; 6 Department of Behavioral and Social Sciences, School of Arts and Sciences, MCPHS University, Boston, MA USA; 7 Hebrew University School of Medicine, Jerusalem, Israel; 8 College of Osteopathic Medicine of the Pacific, Western University of Health Sciences, Lebanon, OR USA; 9 Hebrew University Hadassah Medical School, Jerusalem, Israel; 10 Hadassah Hebrew University Medical Center, Jerusalem, Israel

**Keywords:** Lifestyle Medicine, Student led curriculum, Medical school, Health coaching

## Abstract

**Background:**

By 2020, the World Health Organization predicts that two-thirds of all diseases worldwide will be the result of lifestyle choices. Physicians often do not counsel patients about healthy behaviors, and lack of training has been identified as one of the barriers. Between 2010 and 2014, Hebrew University developed and implemented a 58-h Lifestyle Medicine curriculum spanning five of the 6 years of medical school. Content includes nutrition, exercise, smoking cessation, and behavior change, as well as health coaching practice with friends/relatives (preclinical years) and patients (clinical years). This report describes this development and diffusion process, and it also presents findings related to the level of acceptance of this student-initiated Lifestyle Medicine (LM) curriculum.

**Methods:**

Students completed an online semi-structured questionnaire after the first coaching session (coaching questionnaire) and the last coaching session (follow-up questionnaire).

**Results:**

Nine hundred and twenty-three students completed the coaching questionnaire (296 practices were with patients, 627 with friends /relatives); and 784 students completed the follow-up questionnaire (208 practices were with patients, 576 with friends /relatives). They reported overall that health coaching domains included smoking cessation (263 students), nutrition (79), and exercise (117); 464 students reported on combined topics. Students consistently described a high acceptance of the curriculum and their active role in coaching. Further, most students reported that they were eager to address their own health behaviors.

**Conclusions:**

We described the development and acceptance of a student-initiated comprehensive LM curriculum. Students perceived LM as an important component of physicians’ professional role and were ready to explore it both as coaches and in their personal lives. Thus, medical school deans might consider developing similar initiatives in order to position medical schools as key players within a preventive strategy in healthcare policy.

**Electronic supplementary material:**

The online version of this article (doi:10.1186/s13584-017-0167-y) contains supplementary material, which is available to authorized users.

## Background

By 2020, the World Health Organization predicts that two-thirds of all diseases worldwide will be the result of poor lifestyle choices [1]. In fact, only 21% of U.S. adults meet activity guidelines [2] and nearly the entire U.S. population consumes a diet that is not on par with recommendations [3]. In Israel, only 9.7% of the population engage in the recommended 150 min of physical activity weekly [4] and 26% eat a healthy diet [5]. In response to this gap, Lifestyle Medicine (LM) has been developed and defined as ‘the evidence-based practice of assisting patients and families to adopt and sustain behaviors that can improve health and quality of life’ [6].

Although chronic disease practice guidelines uniformly call for lifestyle change as the first line of therapy [7, 8], physicians often do not counsel patient about healthy behaviors [9, 10]. They do recognize their role in patient health, but report on several barriers to counseling, including a lack of time, reimbursement and training [10, 11]. Indeed, the lack of LM training is widely recognized in U.S. medical schools. Only 27% - provide the 25 h of recommended nutrition education [12], and more than 50% do not have courses that address exercise prescription [13]. Thus LM curricular reform in Undergraduate Medical Education is a logical step to alter the preventive care landscape [6, 14].

Proposals to include LM curriculum in medical schools have been published by several worldwide professional [14, 15], policy [16] and advocacy [17] organizations including the Israeli Ministry of Health [18]. The Bipartisan Policy Center convened a symposium and released a report calling for the inclusion of nutrition and physical activity at all levels of medical education [16]; the US National Physical Activity Plan specifically calls for an increase in physical activity education [17]; and the American Academy of Medical Colleges (AAMC)‘s panel of behavioral and social science experts issued a call to provide rigorous training in social and behavioral sciences in order to equip medical trainees with behavioral and social science-derived knowledge, skills, and attitudes required to practice medicine effectively [15]. Further, in 2013 the Lifestyle Medicine Education (LMEd) Collaborative was founded, which aims to execute these calls, transforming medical education to include LM curricula that addresses critical health behavior domains [19].

Several manuscripts describing LM initiatives in medical schools have recently been published [20, 21]. They typically target a single behavior (e.g. eating, exercising), and describe the program’s impact on students’ attitude, knowledge, and confidence toward prescribing that specific LM domain [20]. There is a limited number of published medical school curricula that describe an integrated approach of various LM topics such as nutrition and exercise together with behavioral change and self-care [22]. These examples include mostly didactic components; however, a few initiatives also include experiential components, in which students are more active and move away from a traditional shadowing learning to active engagement in patient care such as health coaching [23–25] and nutrition education [26].

The process for introducing a new curriculum, including LM [22], often starts with a vision developed by the medical school’s senior management who translates it into teaching modules and supervises its implementation [27]. However, this approach potentially misses important contributions from faculty and medical students [28]. When students suggest an innovation, they usually pursue it in collaboration with an empowered faculty [29], or with support of the students’ association [29, 30]. Interestingly, student-initiated curricula often address health promotion and public health topics in community settings, such as sexual health, or health behavior and prevention counseling [29, 31]. Furthermore, the majority of these curricula include students taking active roles in patient care such as health educators, and physical and mental health supporters to patients and families (i.e. assistance in taking pain medication using home medical equipment, and coping with depression and anxiety) [29, 30].

A key challenge in such innovations, as in any change process, is diffusion into a sustainable curriculum at the medical school [32]. Recent work identifies several factors to promote sustainability of innovations in medical schools. These include stakeholder support, evaluation, institutional and governmental support, peer support, and available resources and funding [33]. Furthermore, to increase the likelihood of sustainability, it has been suggested that medical educators should consider models from the business world [34]. Examples include chartering (boundary setting and team design), learning (discovery and experimentation), mobilizing (garnering resources and building emotional commitment to the initiative) and re-aligning (curricular change) [32]. This report describes the four-year expansion of a student initiative into a comprehensive LM curriculum in our medical school, and it also reports on its level of acceptance by students and faculty.

## Hadassah’s lifestyle medicine curriculum

### Curriculum evolution

During the academic year 2010–2011, a co-author (AS, a medical student at that time) conceived the idea of medical students as health coaches for hospitalized patients and their families, after a teaching moment in an Internal Medicine ward. “*We stood with a faculty by a patient with obesity who smoked (and was hospitalized due to heart failure exacerbation) while his three sons who were obese entered the room with sweet beverages and a cigarette smell… I was afraid that these kids were going to follow the same path as their father…. I asked the faculty about discussing this issue with them, but he thought that it is the community physician’s role…. I didn’t like his answer… I was frustrated because as a student I made a good connection with my patients but I didn’t know how to address these issues…”.* A few days later AS discussed his idea with a senior faculty (MB, School of Public Health) when they met on the stairs - both of them routinely avoiding elevators to increase daily exercise. Together they brought the concept to other faculty, in particular, experts in Sports Medicine (NC) and Nutrition (RP), and students who separately developed the idea of health coaching in the community.

**Fig. 1:**
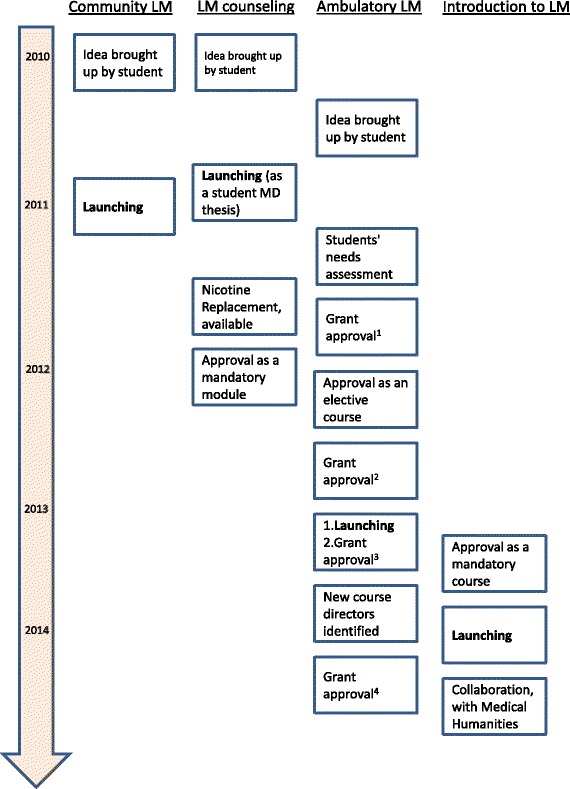
Diffusion Milestones of the Student LM initiative

The diffusion plan of these ideas included 1) establishing a LM steering group with regular meetings to brainstorm, discuss strategy, and monitor progress; 2) surveying students about perceived educational gaps at the medical school curriculum, and sending reports to both the medical school’s and the teaching hospital’s senior managements [35]; 3) advocating the importance of LM education and students’ interest to the Dean’s office and key faculty through meetings and discussions; 4) developing the curriculum gradually, suggesting new components once readiness for additional change was identified; 5) recruitment of community stakeholders such as Health Maintenance Organizations’ senior leadership; 6) including LM topics as part of the Internal Medicine residency program; 7) presenting LM principles to faculty at clinical meetings at the medical school’s affiliated medical centers; and 8) fund raising through advocating the importance of the program to teaching hospitals, community Health Maintenance Organizations, and the medical school’s senior leaderships. Over 4 years, we implemented a comprehensive LM curriculum that addresses various LM domains. The curriculum now includes four courses, which collectively train students in LM throughout their medical school experience: Introduction to LM (first year), Community LM (third year), LM Counseling (fourth year), and Ambulatory LM (fifth and sixth year). Figure 1 presents the curriculum implementation timeline.

### Content development

Four foundational principles guided the development of the curriculum’s content: 1) Each course was directed by one of the program faculty. 2) Each LM domain module (e.g. healthy eating, exercising, smoking cessation, and behavioral change) was developed and consistently supervised by a faculty with expertise in the area. 3) In order to create a spiral curriculum, every year included more advanced applications and opportunities for increased proficiency through iterative practice. Thus, new information and skills were linked directly to learning in previous phases of the spiral [36]. 4) Each course also includes health coaching practice, in which students practice influencing others’ lifestyle behaviors.

Table 1 presents the final 58-h LM curriculum at our medical school for academic year 2013–14. It includes the domains of nutrition, physical activity, smoking cessation and behavioral change spread out into three mandatory courses and one elective. The first two courses (Introduction to LM and Community LM), which are in the preclinical years, are focused on in-class education. The third course (LM Counseling), which is in the inpatient clinical year, is composed of LM teaching rounds that are conducted with each student group on clinical rotations: students present patients who have LM challenges, and program faculty discuss and demonstrate tools for motivational interviewing and coaching. These rounds, led by at least one of the curriculum leaders (NC, RP, MB, AS), were regularly held at the Internal Medicine departments of each of the four teaching hospitals of our medical school. The fourth course (Ambulatory LM, elective), which is community-based, requires students to engage for a full year in coaching one patient in collaboration with his/her family physician. Students present their patient and coaching experience in monthly classes, which are moderated by one of the program faculty and a behavioral change expert, in order to receive feedback and to further discuss LM education.

**Table 1:** Lifestyle Medicine spiral curriculum structure, content areas, methodology, and teachers (as of 2013–2014)

Academic year	Course	Focus of LM Content Areas^a^, *health coaching practice*	Educational Methods	Teachers
1st year (Mandatory)	Introduction to LM (28 h)	LM and health*, short relative/friend coaching practice (elective)*	Lectures; LM history taking,	Sport physician, exercise physiologist, Family Medicine physician, nutritionist, health psychologist, Public Health physician
3rd year (Mandatory)	Community LM (6 h)	LM and public health, *short relative/friend coaching practice (mandatory)*	Lectures, discussions, case studies, webinar	Family Medicine physician, Public Health physician, family physician
4th year (Mandatory)	LM Counseling (4 h)	LM and disease management, *short hospital-based coaching practice (mandatory)*	Bedside teaching, case presentation	Family Medicine physician, Public Health physician
5th–6th year (Elective)	Ambulatory LM (18 h)	LM and disease management, *one-year ambulatory patient coaching practice (elective)*	Case presentation, lectures, hands on workshops	Public Health physician, Family Medicine physician, exercise physiologist, social worker

^a^Content areas include nutrition, physical activity, smoking secession, and behavioral change

Each of the courses includes an assignment to do a health coaching practice with one individual. At least two meetings are required, and at least one of these must be done in person (other meetings can be done remotely through telephone or skype). During the preclinical years, when students do not yet have exposure to patients, the health coaching is conducted with a friend or a relative, and during the clinical years it is with an actual patient. Faculty are available to answer student questions about coaching in the first three courses, and as previously described, the fourth course includes a structured presentation of the coaching practice by the students. In addition to didactics in the courses, students utilize the following tools while completing the coaching practice: 1) A brochure developed for patients about healthy eating, smoking cessation and exercise (translated in four languages) [37]; 2) An on-line module aimed to expand students’ knowledge and skills for behavioral change, motivational interviewing [38] and smoking cessation [39]; and 3) optional nicotine replacement therapy made freely available for patients receiving counseling for smoking cessation.

## Methods

### Setting

The Hadassah Hebrew University Medical School is one of four Israeli medical schools, which offer a medical degree in a 6-year curriculum (composed of three pre-clinical years followed by three clinical years). The medical school has four affiliated teaching hospitals. Except for a mandatory 8-h course in Sports Medicine offered to 5th year students, the curriculum did not include any course on nutrition, behavioral change or other LM domains before the initiation of this program.

### Curriculum evaluations

#### Students’ evaluation

The program evaluation was conducted through coaching practice evaluations which assessed themes of the courses. Students completed an online semi-structured questionnaire after the first coaching session (coaching questionnaire) and the last coaching session (follow-up questionnaire). As validated tools were not available, two online questionnaires were developed and pre-tested by course faculty. These self-report questionnaires assessed the extent to which 1) the coaching was patient-centered, and related to current health conditions; 2) educational tools were utilized and community resources were addressed; 3) follow-up was planned and pursued; and 4) behavioral change occurred.

Open-ended items in both questionnaires included: 1) describe shortly your coaching experience, 2) describe how you felt the individual received your coaching, and 3) state any comment about the entire coaching practice. The follow-up questionnaire also included open-ended items, which asked students to describe to what extent behavioral change occurred. Other items were composed of yes/no questions, and included 1) on-line module usage; 2) brochure distribution; 3) referral to community resources (all in coaching questionnaire); and 4) community resource usage (follow-up questionnaire).

Quantitative data about tools utilization were summarized using Microsoft Excel Software. The open-ended questions in the follow-up questionnaires were analyzed by two researchers and categorized as: 1) behavioral change occurred, 2) behavioral change did not occur, or 3) data are unclear. Further, a qualitative research investigator (AF) mapped and classified students’ open answers in both questionnaires using conventional content analysis [40], where final categories were derived by inductive thematic analysis from the text data [41]. First, all responses were read twice, and a “cherry-pick” was done to questionnaires with rich answers that fit qualitative analysis (600 coaching questionnaires and 124 follow-up questionnaires were picked). Second, the cherry picked responses were read several times to gain a sense of the students’ experiences. Third, words, sentences or paragraphs were marked and grouped into meaning units, until new meaning units no longer appeared. Fourth, the meaning units were condensed, abstracted, labelled, and compared for similarities, differences and overlapping. Some were rich with information so there was a need to divide them into sub-units. Fifth, code words (i.e. words which express in essence the central content of the citations such as feelings, reflections, difficulties) were marked for each meaning unit. Codes were compared for similarities and differences between meaning units. Accordingly, meaning units were rephrased so that each one would be completely different from others. This process continued until saturation (i.e., until there were no overlaps and/or repetitions between the various units). Sixth, the meaning units were grouped into categories, which were as mutually exclusive as possible. A detailed summary explanation was written under each category and a variety of quotes were listed. Last, the final categories were discussed with other members of our team and consensus was reached.

#### Medical school’s faculty evaluation

A 9-item Likert scale questionnaire to evaluate attitudes of our medical school’s teachers about the LM program was developed and pre-tested by the program faculty (Additional file 1: Appendix 1). It was distributed to a purposive sample of 24 Department Chairs, Clerkship Directors, senior physicians and residents in all the Departments of Medicine of the medical school’s teaching hospitals, at the completion of the 3rd year of the curriculum diffusion.

## Results

From 2010 through 2014, 962 students participated in at least one of the curriculum’s LM courses, which included a mandatory coaching practice. Eight hundred and ninety-five (93%) completed the coaching questionnaire (274 with patients, 621with friends/relatives), and 766 (82%) completed the follow-up questionnaire (195 with patients, 571with friends /relatives). In addition, 28 students who did the coaching practice in the elective module completed the coaching questionnaire (22 with patients, 6 with friends/relatives), and 18 completed the follow-up questionnaire (13 with patients, 5 with friends/relatives). Table 2 presents the number and percent of students who reported about including various LM domains in the coaching practices throughout the curriculum.

**Table 2:** – Number of students who completed the coaching practice across the curriculum, and coaching topic domains

	Elective			Mandatory	
	Introduction to LM	LM Counselling	Ambulatory LM	Community LM	LM Counselling
Total # of students	6	17	5	621	274
Smoking succession	2 (33%)	11 (65%)	1 (20%)	152 (24%)	97 (35%)
Healthy nutrition	1 (17%)	0 (0%)	0 (0%)	66 (11%)	12 (4%)
Physical activity	2 (33%)	1 (6%)	2 (40%)	88 (14%)	24 (9%)
Combined	1 (17%)	5 (29%)	2 (40%)	315 (51%)	141 (52%)
Follow-up	NA	13 (76%)	4 (80%)	571 (92%)	195 (71%)

### Student implementation and acceptance of LM curriculum

Four hundred and eighty (45%) students reported on distributing the brochure as part of the coaching, 631 (64%) reported on reviewing the on-line module, and 516 (52%) reported on referring coachees to community resources. Further, 228 (23%) reported that coachees met with these community resources, and 594 (60%) students reported on various degrees of behavioral change of their coachees.

Several categories related to acceptance were reported by the students. Table 3 presents these categories and the lessons learned from each one of them.

**Table 3:** – Categories and lessons learned regarding student acceptance of LM program

Category	Lessons learned
LM in medical education	- LM curriculum is highly accepted by students. - Students perceive LM as an essential component of their future professional role as physicians.
Health coaching practice	- Medical students desire to be actively engaged in patient care. - Mentoring and support should be provided as in all learning of clinical activities. - Students are eager to address their own health behaviors.
Coaching friends and relatives	- Coaching relatives and friends is generally accepted by students as a suitable precursor to coaching patients. - Working with relatives might be too challenging for some students, thus other options such as working with friends should be available. - Curriculum should include specific considerations about working with friends and relatives.
Coaching patients	- Students believe that patients accept their role as health coaches. - Coaching patients motivate students to seek more LM education. - Family physicians recognize the value of coaching their patients by medical students.

#### LM in medical education

Students’ overall acceptance of LM in medical education was high. They appreciated this new area and thought of it as key to medical education and an important factor in physicians’ professional role. Among the open-ended answers, none of the students disputed the importance of this curriculum or the role of physicians in managing their patients’ lifestyle. Further, students saw medical school as a “spring of health” that can impact outer circles in its surrounding community:

*“I think that our goal as physicians is not to cure individuals who come to us with diseases but to help people to be healthy. Education about exercise, smoking, and other aspects of health should start in an early stage of our education”* (coaching questionnaire).

#### Health coaching practice

Students reported that moving from shadow learning to active engagement in care was a positive experience. Students reported being excited about helping others and hearing about the improved behaviors from the people they coached. Further, students also discussed the benefits of the practice and its contribution to their competencies to educate patients:

“*This (e.g. coaching) practice opened my eyes. The concept that I, a medical student without an official credential, can effect a change in my close environment was novel for me. The positive outcomes motivated me to continue helping my family members too…... it broke a psychological barrier to advise patients on healthy lifestyles. When I got to my next advice it was much easier for me to access [to the task] and be more efficient in terms of time”* (follow-up questionnaire).

Some students reported that such a two-session health coaching practice is not enough for a complete lifestyle change:

“…*In order for it to succeed, it has to have a continued supportive framework/program. I think the importance of students here is to raise awareness of the issue and no more. Otherwise it can also provoke antagonism by some of the patients*” (follow-up questionnaire).

In addition to the benefits that individuals who received the coaching gained, several students noted other potential advantages of the practice, primarily its public health potential:

“*This practice is so important! This is preventive medicine in the purest way it could be! During their daily work it is difficult for physicians to counsel… health coaching by medical students who have time, and perceived by patients as having a medical knowledge might bring a meaningful improvement to the preventive medicine landscape”* (follow-up questionnaire).

#### Coaching friends and relatives

Several students reported that, overall, coaching friends and relatives was a positive and joyful experience with advantages for both personal and professional life. Students reported that their friends and relatives had a high commitment to the coaching, and that follow-up was relatively easy. Further, some students stated that coaching friends and relatives offered an opportunity to recruit other family members to the task, to set a family goal, and to have shared goals with their friends/relatives, thus improving their personal behaviors:

*“since my ‘coachee’ is my mom it is easy for me to follow-up”* (follow-up questionnaire); *“I asked my brother to follow-up with my (mother) about the exercise she needs to do when I was absent”* (follow-up questionnaire); “*my mother has started both a new diet and to walk every morning. What amazing is that she is taking my father with her!”* (coaching questionnaire); *“we schedule to run together”* (coaching questionnaire).

However, a number of students also described coaching friends/relatives as an intensive process with difficulties to change the relationship from relatives to provider and coachee:

*“The fact that he is my friend brought more difficulties. I believed the coaching was received seriously and with true honest listening…however as I am not professional or in any official role there was doubt and confusion about my expertise and experience (which I actually feel myself)” (*coaching questionnaire)*.*

#### Coaching patients

Although students generally described satisfaction from coaching a close individual, they were looking forward to coaching individuals whom they did not know, such as hospitalized patients. When they did coach actual patients, most students reported that these sessions progressed smoothly, and that patients accepted their new role in the department. Students also reported that this practice improved their overall relationship with their patients, a connection that usually is not created through a standard intake:

“*She was very grateful for the coaching, and asked where can she leave a thank you letter. I think that the coaching was received in a very positive way”* (coaching questionnaire).

In some cases, students who experienced a short coaching practice with hospitalized patients mentioned doubts about the effectiveness of the coaching:

*“We do not have the tools to effect a meaningful change because we do not have enough knowledge and authority”* (follow-up questionnaire).

However, students who experienced a one-year ambulatory patient coaching practice reported on a satisfying experience with positive outcomes both for themselves and for their patients; such responses were often supported by the patients’ family physicians:

“*Although it wasn’t easy to develop personal relationship and trust with my patient, I think that we found a common language. Thus I could help him formulating walking habits that address his impaired glucose tolerance. During our coaching he lost 8 pounds and his glucose levels have improved. It was a great experience for me on how to develop relationship with patients with complex personality. A task that seems very difficulty in the beginning, but after giving a way part of my concepts as well as addressing his needs, we had good accomplishments… it was an important experience and lesson that I will take to my professional life” (*follow-up questionnaire).

### Acceptance by the medical school faculty

During summer 2013, 24 physicians (Department Chairs, Clerkship Directors, senior physicians, and residents from 6 Internal Medicine wards) received and completed an evaluation. All respondents supported (46%) or very much supported (54%) the program that involved medical students as health coaches. In addition, all the respondents stated that this project was either applicable (54%) or very much applicable (46%) in a teaching hospital setting, and that LM education was either important (33%) or very important (67%). Also, most of responders reported that they either usually (58%) or always (13%) deliver LM education to their patients.

## Discussion

This report describes how students’ LM innovations diffused to form a four-year spiral comprehensive LM curriculum, and on the acceptance level of its implementation in the Hadassah Hebrew University Medical School. Compared to the majority of LM curricula, which predominantly relate to basic sciences [42], this curriculum includes practical knowledge and skills related to patient health behaviors. It also provides the opportunity for students to be actively involved in patient care, and promotes the engagement of the medical school in the health of its surrounding community [43].

The uptake of the LM curriculum by students is in concordance with other studies showing students’ interest in health promotion, and with the emerging literature about students recognizing both the lack of training and the need for a formalized curriculum in LM [44]. Further, not only did students praise the importance of LM, several leveraged their education and addressed their own health behaviors. This aligns with the recommendation of the LMEd Collaborative to add self-care including stress resiliency to nutrition, physical activity, and behavioral change in a LM curriculum [45], and it also supports recent literature that notes the correlation between physicians’ and their patients’ healthy behaviors [46]. Future LM curricula might consider including self-care, especially stress resiliency, as one of their content areas [45].

A key curriculum component that was well accepted by students was the health coaching practice. To date, only a few LM programs have described experiential components, which progress from traditional shadow learning to opportunities for active engagement in patient care. Interestingly, other student driven curricula do include this component [29, 30], and some also include LM topics such as health coaching [23–25] and nutrition education [26]. This report demonstrates how students in clinical years may coach patients after having the experience in pre-clinical years to coach relatives and friends.

Although 60% of students reported on various degrees of behavioral change of their coachees, further development of the role of health coaching practice in medical education is warranted. Factors that students described as important for future success are comprehensive training, mentoring, and collaboration with community resources. Currently the International Consortium for Health and Wellness Coaching is setting requirements and definitions [47]. We recommend further consideration of how medical students might be eligible to become health and wellness coaches with adequate training.

Similar to the development of other curricula [34], the successful diffusion of the innovations in our medical school was enabled by several key factors. These include collaboration between several faculty and students, assessment, stakeholders support, and fund raising. These factors align with all the focus areas identified by the LMEd Collaborative as necessary to facilitate reform in medical schools [45]. Also, it should be noted that another factor that may have contributed to (or enabled) our success was that we approached the medical schools’ leadership with proposed changes that we determined to be realistic and achievable. For example, by recognizing that the pre-clinical years’ curricula do not include interaction with patients, we suggested that students practice coaching in these years with friends or relatives.

In addition to the diffusion of the innovation in our medical school, dissemination to other medical schools also occurred. Indeed, faculty and students from other Israeli medical schools contacted the core group to learn about the initiative, and one medical school has implemented a LM curriculum. Further, an intern rotating at another Israeli academic medical center recently reached out to our group with the support of his chair, asking to import the experience he had as a student in our medical school to his new position. It has been reported that factors that enable curriculum dissemination are educational leadership, personal contacts, rigorous measurement, and attention to implementation science principles [48]. We were able to incorporate two of these factors, i.e. educational leadership and personal contacts, in the dissemination of our innovation to other medical schools.

Both the LMEd Colaborative [45] and the summit on medical school education in sexual health [49] identified students as a key in the creation, diffusion and dissemination of LM educational innovations. Two action items that might improve opportunities for students, both to engage in curricular development and to collaborate with faculty, are to involve students with committees and course debriefing [29] at the local and the national levels. This might also be one of the solutions to the call to increase LM education in medical schools [46]

From our experience a major barrier to implementation was a lack of qualified and dedicated staff. A number of students mentioned that they would have appreciated closer mentoring while pursuing the coaching assignment. Also, there are several limitations to report and issues to address in the future: 1) The long-term sustainability of the curriculum is unknown. Since the completion of this reported period, both positive and negative developments have occurred: Recognizing the importance of LM and the potential of our work, we were awarded a national grant from the Israeli Council for Higher Education to develop an online LM course that will be available to all Israeli academic institutions. Yet, there is currently a shortage of faculty to fully run the program. 2) A more detailed evaluation of the medical school faculty’s acceptance is needed, primarily aimed at understanding how to better incorporate the program into the clinical years. 3) We did not evaluate the patients’ acceptance of the program, and this component could add valuable information regarding the impact of the students’ coaching and potential curricular revisions. 4) A self-care module that includes stress resiliency would greatly contribute to this curriculum

Recently, US primary care residents demonstrated low knowledge of LM and obesity and related management strategies [50]. We aim in the future to follow-up on trainees after completion of medical school in order to assess 1) the degree to which the knowledge barrier has decreased as a result of including LM coaching in their medical school education, 2) the extent of LM education they provide to their patients, and 3) their personal LM behaviors.

Currently, in Israel, most medical schools teach LM, and a LM curriculum for primary care residents was recently piloted [51]. This curriculum might be one answer to address the tsunami of obesity and LM related diseases that students are going to confront in practice [52]. Although medical education is only one component of changing the preventive management landscape, there is an urgent need to create and implement LM curricula in all medical schools. We hope that this report will empower other students and faculty to collaborate with the goal of bringing LM into their medical school curriculum. Training medical students in LM throughout Undergraduate Medical Education can create a new generation of physicians who have the knowledge, skills, and tools to improve and sustain healthy behaviors for themselves and for their patients.

## Conclusions

In conclusion, we described the acceptance by students and faculty of a student-initiated comprehensive LM curriculum, which includes health coaching practice. ‬Students perceived LM as an important component of physicians’ professional roles and they were ready to explore it both as coaches and in their personal lives. Through this early formative and authentic interpersonal human experience, they may become disposed to counsel patients during their postgraduate career. Moreover, the very existence of such a task in the medical school curricula may promote active learning and growth within the faculty and staff to respond to inquiries by students about patient cases. Optimally, both faculty and students will become more collaborative agents of change and advocates for improved health policies in the field of preventive medicine.

## Additional file

Additional file 1: Appendix 1. Faculty Questionnaire. (DOCX 90 kb)

## Abbreviations

LM: Lifestyle medicine
LMEd Collaborative: Lifestyle medicine education collaborative
