# Supplementary material for: Medical students as health coaches: Implementation of a student-initiated Lifestyle Medicine curriculum
Source: Isr J Health Policy Res. 2017 Nov 10;6:42. doi: 10.1186/s13584-017-0167-y (PMC5680812; doi:10.1186/s13584-017-0167-y)
Supplement: Additional file 1: — Appendix 1. Faculty Questionnaire. (DOCX 90 kb) [file 13584_2017_167_MOESM1_ESM.docx]

**Appendix 1 - Faculty Questionnaire**

Faculty/resident, hospital______, department __________, teaching experience (years) _____

During the last few years, a Lifestyle Medicine Curriculum is delivered in the Hebrew University Medical school. It includes 1) training to provide health coaching, 2) a health coaching assignment in which medical student coach inpatients in the Department of Medicine to improve their lifestyle, primarily healthy nutrition, physical activity and smoking cessation, and 3) evaluation. We appreciate your opinion about various component of the curriculum.

1. Are you familiar with the program? (yes/no)
2. Do you think that Lifestyle Medicine topics get enough attention in the Medical School curriculum? (too much attention/ the right amount of attention/ not enough attention/don’t receive any attention)
3. Do you think it is an important topic? (very important/important/slightly important/not important)
4. Do you think it is applicable to use medical students as health promoters at the department? (very applicable/applicable/slightly applicable/not applicable)
5. Will you support such project? (very supportive/supportive/slightly supportive/no supportive)
6. Do you counsel your patients about lifestyle issues during your work in the department? (usually/sometimes/rarely/no)
7. Do you think that the students training for this program is sufficient? (very sufficient/sufficient/slightly sufficient/no sufficient)
8. What should such training include?

- Discussion with a bedside teaching
- Webinar
- Handouts and brochures
- Objective structured clinical examination
- Short course
- Other ________

1. In what academic year this training should be provided (please check any year(s) that apply) (1^st^, 2^nd^, 3^rd^, 4^th^, 5^th^, 6^th^)
